# Anthocyanin Profile, Antioxidant, Anti-Inflammatory, and Antimicrobial against Foodborne Pathogens Activities of Purple Rice Cultivars in Northern Thailand

**DOI:** 10.3390/molecules26175234

**Published:** 2021-08-29

**Authors:** Thanawat Pattananandecha, Sutasinee Apichai, Sasithorn Sirilun, Jakaphun Julsrigival, Kasirawat Sawangrat, Fumihiko Ogata, Naohito Kawasaki, Busaban Sirithunyalug, Chalermpong Saenjum

**Affiliations:** 1 Cluster of Excellence on Biodiversity Based Economics and Society (B.BES-CMU), Chiang Mai University, Chiang Mai 50200, Thailand; thanawat.pdecha@gmail.com (T.P.); sutasinee.apichai@gmail.com (S.A.); jakaphun@gmail.com (J.J.); kasirawat.s@cmu.ac.th (K.S.); 2 Department of Pharmaceutical Sciences, Faculty of Pharmacy, Chiang Mai University, Chiang Mai 50200, Thailand; ssirilun@gmail.com; 3 Center of Excellence for Innovation in Analytical Science and Technology (I-ANALY-S-T), Faculty of Science, Chiang Mai University, Chiang Mai 50200, Thailand; 4 Faculty of Pharmacy, Kindai University, 3-4-1 Kowakae, Higashi-Osaka, Osaka 577-8502, Japan; ogata@phar.kindai.ac.jp (F.O.); kawasaki@phar.kindai.ac.jp (N.K.); 5 Antiaging Center, Kindai University, 3-4-1 Kowakae, Higashi-Osaka, Osaka 577-8502, Japan

**Keywords:** purple rice, anthocyanin content, antioxidant, anti-inflammatory, anti-foodborne pathogens

## Abstract

Five glutinous purple rice cultivars and non-glutinous purple rice cultivated in different altitudes in the north of Thailand were collected. The samples were extracted using ethanol and determined for anthocyanins using HPLC. The total phenolic content (TPC), total flavonoid content (TFC), and the antioxidant, anti-inflammatory, and antimicrobial activities against foodborne pathogens were investigated. The highland glutinous cultivar named Khao’ Gam Luem-Phua (KGLP) extract had significantly high levels of cyanidin 3-*O*-glucoside, peonidin 3-*O*-glucoside, delphinidin 3-*O*-glucoside, TPC, and TFC, as well as exerting a potent antioxidant activity through ABTS assay (524.26 ± 4.63 VCEAC, mg l-ascorbic-ascorbic/g extract), lipid peroxidation (IC_50_ = 19.70 ± 0.31 µg/mL), superoxide anions (IC_50_ = 11.20 ± 0.25 µg/mL), nitric oxide (IC_50_ = 17.12 ± 0.56 µg/mL), a suppression effect on nitric oxide (IC_50_ = 18.32 ± 0.82 µg/mL), and an inducible nitric oxide synthase production (IC_50_ = 23.43 ± 1.21 µg/mL) in combined lipopolysaccharide-interferon-γ-activated RAW 264.7 murine macrophage cells. Additionally, KGLP also exhibited antimicrobial activity against foodborne pathogens, *Staphylococcus aureus*, *Escherichia coli*, *Salmonella* Enteritidis, and *Vibrio parahaemolyticus*. These results indicate that Thai glutinous purple rice cultivated on the highland could be a potent natural source of antioxidants, anti-inflammatories, and antimicrobial agents for use as a natural active pharmaceutical ingredient in functional food and nutraceutical products.

## 1. Introduction

Free radicals are an atom or molecule which contain one or more unpaired electrons in outer orbital and are strongly reactive to macromolecules including lipids, carbohydrates, DNA, and proteins [1]. This is partly due to oxidative stress, which is the adverse effect of oxidants on physiological or biological function and induced compounds such as metal and toxic compounds [2]. Reactive oxygen species (ROS) and reactive nitrogen species (RNS) play important roles in various biological systems by the overproduction of reactive species, namely hydrogen peroxide (H_2_O_2_), hydroxyl radical (^•^OH), superoxide anions (O_2_^•–^), peroxyl (ROO^•^), lipid peroxyl radical (LOO^•^), nitric oxide (NO), and peroxynitrite (ONOO^−^) [1]. They are involved in various imbalances and disorders including aging, hypertension, diabetes mellitus, atherosclerosis, inflammatory-related diseases, ischemic diseases, neurodegenerative disorders, respiratory diseases, and cancer [1,3]. Antioxidants are thought to be highly effective in scavenging and managing both ROS- and RNS-mediated functions. They act as radical scavengers converting to a less reactive species and inhibit the oxidation of biomolecules by inhibiting the initiation or propagation step of oxidative chain reactions [4]. Antioxidants can be produced to fight against free radicals both in vivo by the body, including glutathione, ubiquinone, superoxide dismutase, catalase, etc., or taken from food and medicinal plants [3,5]. Antioxidants from plants are mainly polyphenols, flavonoids, carotenoids, and vitamins [6].

There are natural antioxidants found from bioresources, plants, medicinal plants, and metabolites produced from various microbials that are currently used as natural active pharmaceutical ingredients (NAPIs) and nutraceuticals to improve human health [7,8], such as antioxidant, anti-inflammatory, antibacterial, anti-aging, and anticancer [9]. Thai purple rice (*Oryza sativa* L. *indica*) is a native plant cultivated in northern Thailand. Purple rice contains a higher level of protein, vitamins, minerals, and bioactive compounds comparable to white rice. However, the level of bioactive compounds vary on the location of plantation and cultivar [10]. Purple rice has been considered as a functional food and nutraceutical for health which has been widely consumed since ancient times in Asian countries, especially in northern Thailand. It has been reported as a potent source of antioxidants and as having health-promoting compounds [11]. The essential bioactive appraised polar and non-polar components in the rice include phenolic acids, flavonoids, anthocyanins, sterols, tocopherols, tocotrienols, and γ-oryzanol [12]. These compounds have been indicated, with various biological activities, to modulate and improve diseases in human health such as oxidative diseases and cancer [13]. It is well known that purple rice contains higher nutritional and functional properties than other pigmented rice. Consistent with other Asian countries, Thailand also grows and consumes a large amount of rice. More than 5000 rice varieties are known within the country, which include purple rice cultivars. Therefore, this work is aimed at finding the appropriate fractions of purple rice sources of phytochemicals, based on biological components and biological activities, for a nutraceutical development.

The aim of this study is to prove and support native northern Thai purple rice, cultivated in different altitudes, as a strong natural antioxidant source for the prevention of oxidation radicals with in vitro antioxidant standard models. The current study also analyzes the anthocyanin profile and content, and conducts a cell-based study for anti-inflammatory activity and antimicrobial activity against foodborne pathogens. The potent antioxidation, anti-inflammatory, and antimicrobial activities might be the potential candidate to be used as the natural active pharmaceutical ingredients (NAPIs) for the prevention of several acute and chronic diseases in functional food and nutraceutical products.

## 2. Results and Discussion

### 2.1. Anthocyanin Content 

Anthocyanins have been shown to lower the risk of severe illnesses including cancer and obesity, as well as to have antiviral, anti-inflammatory, and anti-aging properties [14]. They are present in the outer grain layers whilst the pericarp and aleurone layers contain the majority of grain anthocyanin, accounting for 85% of the total grain content [15]. Anthocyanin concentration is derived directly from the production of the purple/black pigment [16]. In this study, three anthocyanins including cyanidin 3-*O*-glucoside (C3G), peonidin 3-*O*-glucoside (P3G), and delphinidin 3-*O*-glucoside (D3G) in purple rice extracts were analyzed, and the results are presented in Table 1. C3G was the main anthocyanin content in purple rice extract, followed by P3G and D3G. Khao′ Gam Leum-Phua (KGLP) had the highest (*p* < 0.05) of C3G and D3G at 55.26 ± 0.71 and 1.95 ± 0.13 mg/g extract, while Khao′ Gam Pah E-Kaw (KGPEK) had the highest (*p* < 0.05) amount of P3G at 15.91 ± 0.47 mg/g extract. Interestingly, D3G was only detected in the highland rice, (KGLP and KGPEK extracts), ranging from 1.55 ± 0.12–1.95 ± 0.13 mg/g extract. Several studies have shown that rice grains that seem consistently dark in color have more significant amounts of anthocyanin [17,18]. The nutritional content of northern Thai purple rice cultivated at various altitudes (highland and lowland) was investigated. In the highland, particular genotypes produce rice with more intense pigment and more significant monomeric anthocyanin contents, whereas only some did so in the lowland [19]. Twenty-five rice brans from 25 rice varieties were examined for anthocyanins contents. The results showed cyanidin-3-glucoside (82.3%) was the major anthocyanin identified, followed by peonidin-3-glucoside (14.6%), cyanidin-3-galactoside (1.2%), cyanidin-3-rutinoside (1.0%), cyanidin (0.7%), and peonidin (0.2%), respectively [17]. Whole grain KGPEK, KGDSK, and KHN were reported to contain C3G and P3G at 88 and 32, 50 and 48, also 133 and 20 mg/100 g dried samples, respectively [20,21,22]. Moreover, KGLP and KHN rice bran were reported to contain C3G and P3G at 2277 and 792, and also 926 and 1422 mg/100 g samples, respectively [23]. D3G was recently detected in a small amount in KGDSK [24]. Several studies have shown that glutinous endosperm types have higher anthocyanin concentrations than non-glutinous rice varieties. Eight glutinous rice samples from Thailand gathered anthocyanins ranging from 81 to 442 mg/100 g, whereas five non-glutinous rice samples gathered anthocyanins ranging from 21 to 85 mg/100 g [25]. Similarly, anthocyanin contents in four glutinous and four non-glutinous rice types ranged from 42 to 271 mg/100 g and 12 to 40 mg/100 g, respectively [26]. Evidence suggests that the anthocyanin content in purple rice significantly increases in plantation altitude. Thus, while most purple rice cultivars are traditional/local rice representing variations across areas and growth environments (glutinous and non-glutinous rice types and upland and lowland rice types), anthocyanin concentration in the grain varies [19,27,28]. 

### 2.2. Total Phenolic and Total Flavonoid Content

Rice contains phenolic acids, flavonoids, anthocyanins, and procyanidins, among other phenolic substances. Non-pigmented rice cultivars typically contain only phenolic acids, while pigmented rice contains more polyphenol chemicals [13]. The total phenolic content (TPC) and total flavonoid content (TFC) of rice extract were expressed as gallic acid equivalents (GAE) and quercetin equivalent (QE), as illustrated in Table 2. Significant differences in TPC and TFC were observed among the five rice cultivars. KGLP was shown to dominate significantly highest (*p* < 0.05) TPC and TFC at 595.53 ± 7.36 mg GAE/g extract and 379.35 ± 4.26 mg QE/g extract, followed by KGPEK with 570.49 ± 6.53 mg GAE/g extract of TPC and 340.24 ± 3.64 mg QAE/g extract of TFC. While the lowest TPC was found in KHN and the lowest TFC was found in KGDSK and Khao′ Niaw Dam (KND).

The results corresponded to anthocyanin contents in Section 2.1. Highland rice extracts (KGLP and KGPEK) had a higher TPC and TFC content than lowland rice extracts (KGDSK, KND, and KHN). Additionally, the glutinous rice extracts (KGLP, KGPEK, KGDSK, and KND) had higher TPC and TFC than those of non-glutinous rice extract (KHN). TPC and TFC were higher in KGLP than in KGDSK and KHN, similar to the previous research [25]. Peanparkdee et al. [23] studied phenolic content, anthocyanin content, and antioxidant activity of ethanolic purple rice bran extracts from Thai rice cultivars. The results showed glutinous purple rice bran extracts KGLP had higher TPC than non-glutinous purple rice bran extract KHN. However, TFC in KHN rice bran extract seems to be higher than in KGLP rice bran extract. The predominant phenolic compounds found in red and black rice grains were protocatechuic acid, vanillic acid, syringic acid, p-coumaric acid, ferulic acid, and isoferulic acid [29], while anthocyanin is the most abundant flavonoid in pigmented rice. In addition, the flavonols namely kaempferol and quercetin were found to be the most abundant, whereas apigenin was the most abundant flavone component in pigment rice [30].

### 2.3. Antioxidant Assay

**Figure 1:**
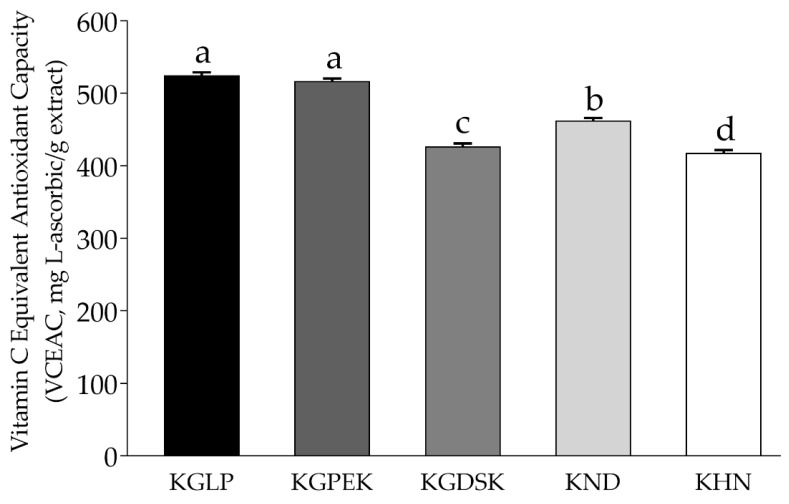
ABTS radical scavenging activity of purple rice extracts. The values are expressed as mean ± SD (*n* = 3). Different superscript letters indicate significant difference at *p* < 0.05. VCEAC: Vitamin C equivalent antioxidant capacity.

The ABTS assay for evaluating antioxidant capacity has become well-known in determining extracts from both biological and chemical synthesis substances [31]. Lipid peroxidation, superoxide anion, and nitric oxide assay are also comprehensive analyses to determine the antioxidant capacity of plants extract. Figure 1 showed KGLP and KGPEK exhibited significantly highest (*p* < 0.05) radical scavenging activity on ABTS^•+^, with the VCEAC value of 524.26 ± 4.63 and 516.31 ± 4.18- mg l-ascorbic/g extract, respectively. These results corresponded with the previous study by Pramai and Jiamyangyuen [25] who determined ABTS^•+^ scavenging activity in rice varieties cultivated in a different location in the north of Thailand and reported that KGLP had a significantly higher ABTS^•+^ scavenging activity than KGDSK and KHN.

Interestingly, Table 3 shows KGLP had the significantly highest (*p* < 0.05) inhibition effect on lipid peroxidation and nitric oxide scavenging activity, with the IC_50_ values of 19.70 ± 0.31 and 17.12 ± 0.56 (µg/mL), and the values were not significantly different when compared to quercetin, which was used as the positive control. Moreover, both KGLP and KGPEK had the significantly highest (*p* < 0.05) superoxide anion scavenging activity, with IC_50_ of 11.20 ± 0.25 and 11.96 ± 0.65 (µg/mL), respectively.

There was a remarkable correlation between the relatively high among of TPC, TFC, cyanidin 3-*O*-glucoside, peonidin 3-*O*-glucoside, delphinidin 3-*O*-glucoside, and ABTS^•+^ scavenging activity with r^2^ 0.960, 0.935, 0.893, 0.826, and 0.916; *p* < 0.01, respectively, as shown in Supplementary Materials Table S1. These results can indicate that the antioxidant capacities on ABTS^•+^, nitric oxide, and superoxide anion scavenging activities, and also the inhibition effect on lipid peroxidation, have correlated with anthocyanins, TPC, and TFC in purple rice extracts. Similarly to the study by Ngamdee et al. [32], five black glutinous rice cultivars from Thailand, including the KGDSK cultivar, were investigated as to their total anthocyanin content (TAC), TPC, TFC, and antioxidant activity against nitric oxide, superoxide anion, and lipid peroxyl radicals. They concluded that the ratio of TAC, TFC, and TPC correlated with the extracts and antioxidant activities. Phenolic and flavonoid compounds act as reducing agents, free radical scavengers, and quenchers of singlet oxygen formation. Pigmented rice cultivars showed high antioxidant capacity along with their highest flavonoid and polyphenol content [33]. According to a review of 316 publications, free radical scavenging activities are sensitive to the presence of TPC and TFC in the samples. The genetic composition of various rice varieties, preharvest variables, storage conditions, and analysis methods are also the main reasons responsible for the significant variance in antioxidant content [34].

### 2.4. Anti-Inflammatory Activities Assay

The inhibitory effects of purple rice extract on nitric oxide and iNOS production in combined lipopolysaccharide (LPS)-Interferon-*γ* (IFN-*γ*) activated RAW 264.7 cells are shown in Table 4. KGLP exerted the highest (*p* < 0.05) inhibitory effect on both cellular NO and iNOS with the IC_50_ value at 18.32 ± 0.82 and 23.43 ± 1.21 µg/mL, respectively, without exerting cytotoxicity. Interestingly, there was a correlation between the relatively high among of TPC, TFC, cyanidin 3-*O*-glucoside, peonidin 3-*O*-glucoside, and the inhibition effects on NO and iNOS production in RAW 264.7 cells induced by combined LPS-IFN-γ as shown in Table S1. Additionally, the results showed a similar inhibitory effect trend as radical scavenging activity. According to this rationale, the northern Thai purple rice extract had a high content of polyphenolic compounds and anthocyanin, which are linked to antioxidant and anti-inflammatory activities.

C3G and its metabolites extracted from black rice have been reported to inhibit the production of the proinflammatory cytokines including tumor necrosis factor-alpha (TNF-α), interleukin-1beta (IL-1β), the inflammatory mediators (NO and prostaglandin E2), and the gene expression of iNOS and cyclooxygenase-2 (COX-2) in lipopolysaccharide (LPS)-induced RAW 264.7 cells [35]. Eight flavonoids have been shown to inhibit the activation of nuclear factor-κB (NF-κB) which is a significant transcription factor for iNOS. Moreover, the activation of the signal transducer and activator of transcription 1 (STAT-1) was likewise suppressed by genistein, kaempferol, quercetin, and daidzein [36]. Five purple rice bran extracts exhibited a significant anti-inflammatory action through an inhibitory impact on NO generation in combined LPS-IFN-*γ*-activated RAW 264.7 murine macrophage cells [37]. Thai pigmented rice cultivars namely Khao’ Gam Muang with a high content of TAC and total proanthocyanidins exerted a potent inhibitory effect on cellular NO and iNOS activity [38].

### 2.5. Antimicrobial Activity against Foodborne Pathogens

**Figure 2:**
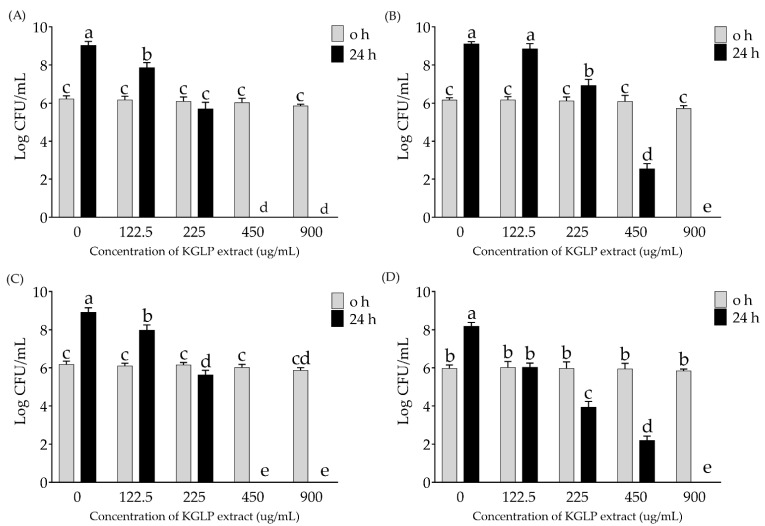
Antimicrobial activity of KGLP extracts on foodborne pathogens. (**A**) *S. aureus*, (**B**) *E. coli*, (**C**) *S.* Enteritidis, and (**D**) *V. parahaemolyticus*. The values are expressed as mean ± SD (*n* = 3). Different superscript letters indicate significant difference at *p* < 0.05.

Foodborne diseases have a significant public health impact, causing morbidity and mortality globally. KGLP extract was selected to determine its antimicrobial potential on the major strains that cause foodborne outbreaks, including *S. aureus*, *E. coli*, *S.* Enteritidis, and *V. parahaemolyticus*. The determination was performed by comparing the viable cell count (log CFU/mL) of the pathogens at 0 and 24 h cultivated with various concentrations of KGLP extract. The results are shown in Figure 2; the antimicrobial activity was correlated with the concentrations of KGLP used in the experiment. KGLP at 450 and 900 µg/mL significantly reduced (*p* < 0.05) *S. aureus* and *S.* Enteritidis with 100% reduction at 24 h of incubation period while *E. coli* and *V. parahaemolyticus* was completely inhibited with 900 µg/mL of KGLP.

Purple rice extract, KGLP, showed its antimicrobial ability to inhibit foodborne pathogens including *S. aureus*, *E. coli*, *S.* Enteritidis, and *V. parahaemolyticus.* Especially on *S. aureus* and *S.* Enteritidis that exerted 100% reduction. Anthocyanins extracted from various plants were studied for their antimicrobial properties against pathogens. Polyphenols may interact with bacterial membrane proteins in both hydrophobic and hydrogen bonding ways. They may sequester essential ions for protein stability and donate or accept electrons across the membrane interface, rendering them versatile antibacterial agent [39,40,41,42]. Evidence showed that antimicrobial efficacy is dose-dependent on anthocyanin content [39]. Gram-positive bacteria is more sensitive to the effects of anthocyanins than gram-negative bacteria. Both membrane and intracellular interactions of these molecules have a role in anthocyanin activity. Moreover, multiple mechanisms and synergies are believed to be involved in the antimicrobial action of anthocyanin-rich plants [43]. We proposed that a part of the antibacterial mechanism of anthocyanin, caused from the positive charge at the oxygen atom of the C-ring, might interact with the negative charge of phosphate in the bacterial cell wall, which destabilizes the cell wall and interferes with osmosis. Current results indicated that the potential of purple rice extract to be used as a natural preservative to inhibit the growth of foodborne pathogens.

## 3. Materials and Methods

### 3.1. Preparation of Purple Rice Extract

Five native northern Thai purple rice, including Khao’ Gam Luem-Phua (KGLP, Phop Phra Agriculture Office, Tak, Thailand), Khao’ Gam Pah E-Kaw (KGPEK, Mae Hong Son Rice Research Center, Mae Hong Son, Thailand) Khao’ Gam Doi Saket (KGDSK, Chiang Mai Rice Research Center, San Pa Tong District, Chiang Mai, Thailand), Khao’ Niaw Dam (KND), and Khao’ Hom nil (KHN, Mae Rim District, Chiang Mai, Thailand), had their husks removed and were then stored at −20 °C until use. The rice samples were extracted with ethanol pH 2.0 at the ratio 1:10 at 60 °C for 120 min, with shaking at 180 rpm. The crude extracts were filtrated, evaporated using a rotary evaporator under reduced pressure, and dried in vacuum dryer. The details of the purple rice samples are shown in Table 5.

### 3.2. Determination of Anthocyanins by HPLC

Anthocyanins extracted with ethanol and lactic acid in both glycoside and glycine form were quantified with reverse phase HPLC by the method from Saenjum et al. [44]. Briefly, The HPLC System was equipped with SymmetryShield^®^ C-18 column (4.6 × 250 mm) and used a multiwavelength detector. The column was eluted with a linear gradient mobile phase operated from 0 to 40 min, with acetonitrile ranging from 10% to 20% at a flow rate of 1.0 mL/min. The separated anthocyanins were detected and measured at 520 nm.

### 3.3. Determination of Total Phenolic Content

The total phenolic content of the tested sample was determined by the Folin–Ciocalteu reagent following a slightly modified method of Saenjum et al. [45]. Briefly, 100 μL of the tested sample (1 mg/mL) and positive control, gallic acid, were made up to 2 mL with deionized water, mixed thoroughly with 100 μL of Folin–Ciocalteu reagent, followed by the addition of 300 μL of 20% (*w*/*v*) sodium carbonate. The mixture was incubated at room temperature for 10 min in the dark and absorbance was measured at 725 nm using a UV/VIS spectrophotometer. The total phenolic content was calculated from the calibration curve and the results were expressed as mg of gallic acid equivalent (GAE) per g dry extract.

### 3.4. Determination of Total Flavonoid Content

The total flavonoid content of the tested sample was determined using a modification from Shen et al. [46]. Briefly, an aliquot (1 mL) of the tested sample or positive control, quercetin, was mixed with 4 mL of ethanol and then 0.3 mL of 5% NaNO_2_ solution; 0.3 mL of 10% AlCl_3_ solution was added after 5 min of incubation. The mixture was allowed to stand for 5 min. Then, 2 mL of 1 M NaOH solution was added and the final volume of the mixture was brought up to 10 mL with ethanol. The solution was mixed and allowed to stand for 10 min and absorbance was measured at 510 nm. The total flavonoid content was calculated from a calibration curve and the results were expressed as mg quercetin equivalents (QE) per g dry extract.

### 3.5. Antioxidant Assay

#### 3.5.1. ABTS Assay

The ABTS (2,2′-Azino-bis(3-ethylbenzothiazoline-6-sulfonic acid)) free radical cation decolorization of the tested sample was determined using the method described by Saenjum et al. [45]. Briefly, ABTS^•+^ was generated by the oxidation of 7.0 mM ABTS with 2.5 mM potassium persulfate. The ABTS^•+^ was mixed with the tested sample at different concentrations (10 to 1000 µg/mL), comparing with the positive control l-ascorbic acid, then incubated at room temperature for 5 min. The color reaction was measured at 734 nm spectrophotometrically. The result of ABTS^•+^ decolorization was expressed as gram of vitamin C equivalent antioxidant capacity (VCEAC) per g tested sample.

#### 3.5.2. Lipid Peroxidation Assay

The inhibition on linoleic acid peroxidation was determined using the modified from Choi et al. [47]. The reaction mixtures were composed of Tris-HCl, linoleic acid emulsion, l-ascorbic acid, and tested sample or positive control, quercetin and C3G. The reaction was induced by the addition of ferrous sulfate and incubated at 37 °C for 30 min. The reaction was stopped by adding trichloroacetic acid (TCA). Then, 100 µL of thiobarbituric acid was added to an aliquot mixed reaction of 500 µL, heated at 95 °C for 10 min, and then cooled on an ice bath. The solution was centrifuged at 3500 rpm for 10 min. The supernatant was collected and absorbance was measured at 540 nm. IC_50_ (50% inhibition concentration) was calculated and compared with positive control. 

#### 3.5.3. Superoxide Anion Scavenging Activity Assay

Superoxide anion scavenging activity was determined using the non-enzymatic phenazine methosulfate-nicotinamide adenine dinucleotide (PMS/NADH) system by oxidation of NADH and assayed by the reduction of nitro blue tetrazolium (NBT). The reaction was generated in 200 µL of phosphate-buffered saline (PBS) at pH 7.4 containing 2.5 µM NADH, 0.5 µM NBT, 2.5 µM EDTA, and tested sample or positive control, quercetin, C3G, and l-ascorbic acid, at different concentrations prepared in a 96-well plate. PMS was added to initiate the reaction and incubated at room temperature for 5 min. Then, the reaction mixture was measured spectrophotometrically at 560 nm and calculated for IC_50_ compared to positive control.

#### 3.5.4. Nitric Oxide Scavenging Activity Assay

Nitric oxide scavenging activity was generated by sodium nitroprusside (SNP) interacting with oxygen, then the produced nitrite ions were measured by Griess reaction [45]. The reaction mixture consisted of 800 μL of sodium nitroprusside and different concentrations of tested samples or the positive control, namely quercetin, C3G, and curcumin in the volume of at 200 μL. Then, the reaction mixture was incubated at 37 °C for 150 min. After incubation, the reaction mixtures were mixed with Griess reagent. The absorbance of the chromophore formed during diazotization of nitrite with Griess reagent was measured at 540 nm and calculated for IC_50_ compared to positive control.

### 3.6. Determination of Anti-Inflammatory Activities

The anti-inflammatory activities of purple rice extracts were investigated through the inhibitory effect on nitric oxide (NO) and inducible nitric oxide synthase (iNOS) production in LPS-IFN-*γ*-activated RAW 264.7 cells using the modified method of Sirithunyalug et al. [48]. Briefly, RAW 264.7 cells were cultured in Dulbecco’s modified Eagle’s medium (DMEM) supplemented with 10% fetal bovine serum (FBS), 100 units/mL penicillin, and 100 µg/mL streptomycin. RAW 264.7 cells were pre-incubated in 24-well plates for 24 h. Then cells were given a fresh medium containing various concentrations of the tested samples and positive control, curcumin, quercetin, and C3G. After 12 h of incubation period, the combined LPS and IFN-*γ* were added and incubated for 72 h. Then, the culture medium supernatants were collected to analyze for nitric oxide using Griess reagent as an indicator of nitric oxide production. The absorbance was measured at 540 nm against a standard curve of potassium nitrite [20]. Fresh culture medium was used as a blank. Additionally, the cells were lysed to yield cell lysates and measure the production of iNOS in the cell lysates using a commercially available mouse iNOS ELISA kit. At the same time, the protein produced by RAW 264.7 cells were analyzed using Bradford reagent (Merck, Germany) [21]. Concurrently, the viability of RAW 264.7 cells was assayed according to the improved method of Jomha et al. [22] and Saenjum et al. [10], with slight modifications. The effect of tested sample and positive control on cell viability was assayed after stimulation with combined LPS-IFN-*γ* in the absence or presence of purple rice extracts for 72 h using the cell proliferation reagent PrestoBlue™ (Invitrogen, Waltham, MA, USA).

### 3.7. Determination of Antimicrobial Activity against Foodborne Pathogens

Anthocyanin-enriched extract prepared from KGLP was selected to determine antimicrobial activity against foodborne pathogens according to the highest anthocyanin content, antioxidant, and anti-inflammatory activities. The tested sample was diluted in the corresponding broth. *S. aureus* ATCC 25923, *E. coli* ATCC 25922, *S.* Enteritidis ATCC 14028, and *V. parahaemolyticus* ATCC 17802 were used following the modified method of Sun et al. [39] and Pelyuntha et al. [49]. A total of 200 μL of each strain inoculum was mixed to the different concentrations of the tested sample (1800 μL), the final concentrations of the tested samples were 112.5, 225, 450, and 900 μg/mL. The negative control was a mixture of medium broth and tested samples without any tested pathogens, while positive control was established by combining medium broth and each tested pathogen. All tested tubes were incubated with shaking at 180 rpm at 37 °C for 24 h. Then, the viable cells were comparably counted on tryptic soy agar (TSA) at 0 and 24 h, whereas the viable cells for *V. parahaemolyticus* were counted on TSA supplemented with 3% NaCl.

### 3.8. Statistical Analysis

SPSS software (version 17.00) was used to analyze all the data statistically. A one-way ANOVA was used for finding any significant difference between treatments, *p* < 0.05 was considered to be significant, and further significance between groups was analyzed using a Duncan post hoc test. Results are presented as the mean ± standard deviation of 3 independent experiments.

## 4. Conclusions

In summary, among the purple rice varieties, anthocyanins content (C3G, P3G, and D3G), TPC, TFC, antioxidant, and anti-inflammatory activities of highland rice glutinous rice varieties, namely KGLP and KGPEK, were higher than those of lowland rice varieties (KGDSK and KND) and the non-glutinous rice variety (KHN). The results also showed the relationship between the phytochemical contents and their antioxidant and anti-inflammatory activities. Our findings suggested that the purple rice extracts from highland glutinous rice cultivars, KGLP and KGPEK, are potent rice cultivars that contain high anthocyanins, TPC, and TFC, as well as exhibiting high inhibitory activity against free radicals (ABTS^•+,^ lipid peroxyl radical (LOO^•^), superoxide anions (O_2_^•−^), nitric oxide (NO), and also suppressed proinflammatory mediators (NO and iNOS). Moreover, KGLP also exerted antimicrobial activity against foodborne pathogens, *S. aureus*, *E. coli*, *S.* Enteritidis, and *V. parahaemolyticus*. Therefore, purple rice cultivars could be used to boost the nutritional content and efficacy of food supplements, functional food, and nutraceutical products. 

## Figures and Tables

**Table 1:** Anthocyanin content of purple rice extracts.

Samples	Cyanidin 3-*O*-Glucoside (mg/g Extract)	Peonidin 3-*O*-Glucoside (mg/g extract)	Delphinidin 3-*O*- Glucoside (mg/g Extract)
KGLP	55.26 ± 0.71 ^a^	14.24 ± 0.46 ^b^	1.95 ± 0.13 ^a^
KGPEK	52.20 ± 0.89 ^b^	15.91 ± 0.47 ^a^	1.55 ± 0.12 ^b^
KGDSK	29.62 ± 0.80 ^c^	10.48 ± 0.38 ^c^	ND
KND	28.91 ± 0.74 ^cd^	10.70 ± 0.40 ^c^	ND
KHN	27.04 ± 0.68 ^d^	8.54 ± 0.31 ^d^	ND

The values are expressed as mean ± SD (*n* = 3). Different superscript letters in the same column indicate a significant difference at *p* < 0.05. KGLP: Khao′ Gam Leum-Phua, KGPEK: Khao′ Gam Pah E-Kaw, KGDSK: Khao′ Gam Doi Saket, KND: Khao′ Niaw Dam and KHN: Khao′ Hom Nil. ND: Not detected.

**Table 2:** Total phenolic and flavonoid contents of purple rice extracts.

Samples	Total Phenolic Content (TPC) (mg GAE/g Extract)	Total Flavonoid Content (TFC) (mg QE/g Extract)
KGLP	595.53 ± 7.36 ^a^	379.35 ± 4.26 ^a^
KGPEK	570.49 ± 6.53 ^b^	340.24 ± 3.64 ^b^
KGDSK	489.39 ± 5.16 ^d^	291.93 ± 3.99 ^d^
KND	533.91 ± 5.54 ^c^	323.21 ± 4.74 ^c^
KHN	451.81 ± 4.85 ^e^	286.40 ± 3.82 ^d^

The values are expressed as mean ± SD (*n* = 3). Different superscript letters in the same column indicate a significant difference at *p* < 0.05. KGLP: Khao′ Gam Leum-Phua, KGPEK: Khao′ Gam Pah E-Kaw, KGDSK: Khao′ Gam Doi Saket, KND: Khao′ Niaw Dam and KHN: Khao′ Hom Nil.

**Table 3:** IC_50_ on inhibition effect on lipid peroxidation, nitric oxide, and superoxide anion scavenging activities.

Samples/ Positive Control		IC_50_ (µg/mL)	
	Lipid Peroxidation	Superoxide Anion	Nitric Oxide
KGLP	19.70 ± 0.31 ^c^	11.20 ± 0.25 ^c^	17.12 ± 0.56 ^c^
KGPEK	21.45 ± 0.38 ^b^	11.96 ± 0.65 ^c^	18.81 ± 0.33 ^b^
KGDSK	21.62 ± 0.37 ^b^	14.78 ± 0.30 ^ab^	19.38 ± 0.38 ^b^
KND	24.57 ± 0.35 ^a^	14.05 ± 0.31 ^b^	21.49 ± 0.34 ^a^
KHN	25.00 ± 0.31 ^a^	15.60 ± 0.45 ^a^	22.31 ± 0.36 ^a^
Quercetin	19.57 ± 0.42 ^c^	9.40 ± 0.37 ^d^	17.54 ± 0.30 ^c^
Cyanidin-3-*O*-glucoside	16.64 ± 0.38 ^d^	9.55 ± 0.34 ^d^	13.76 ± 0.28 ^d^
l-ascorbic acid	ND	7.55 ± 0.31 ^e^	ND
Curcumin	ND	ND	6.68 ± 0.28 ^e^

All values are expressed as mean ± standard deviation (*n* = 3). Different letters in each method indicate a significant difference (*p* < 0.05). ND = Not determined.

**Table 4:** IC_50_ on inhibition effect on nitric oxide and iNOS production.

Samples/ Positive Control	IC_50_ (µg/mL)	
	Nitric Oxide	iNOS
KGLP	18.32 ± 0.82 ^d^	23.43 ± 1.21 ^d^
KGPEK	20.34 ± 0.98 ^cd^	24.66 ± 0.87 ^c^
KGDSK	24.50 ± 0.97 ^b^	29.43 ± 0.98 ^ab^
KND	22.54 ± 0.80 ^bc^	27.94 ± 1.17 ^b^
KHN	29.66 ± 0.91 ^a^	31.74 ± 1.32 ^a^
Quercetin	15.86 ± 0.67 ^e^	20.61 ± 1.18 ^d^
Curcumin	12.61 ± 0.74 ^f^	14.70 ± 0.91 ^e^
Cyanidin-3-glucoside	13.48 ± 0.85 ^ef^	16.68 ± 0.92 ^e^

All values are expressed as mean ± standard deviation (*n* = 3). Different letters in each method indicate a significant difference (*p* < 0.05).

**Table 5:** Varieties, type, altitude, and growing location of rice samples.

Rice Sample	Abbreviation	Type	Altitude	Growing Locations
Khao’ Gam Luem-Phua	KGLP	Glutinous	Highland	Tak
Khao’ Gam Pah E-Kaw	KGPEK	Glutinous	Highland	Mae Hong Son
Khao’ Gam Doi Saket	KGDSK	Glutinous	Lowland	Chiang Mai
Khao’ Niaw Dam	KND	Glutinous	Lowland	Chiang Mai
Khao’ Hom nil	KHN	Non-glutinous	Lowland	Chiang Mai

## Data Availability

The original contributions generated for this study are included in the article; the data presented in this study are available on request from the corresponding author.

## Supplementary Materials

The following are available online, Table S1: Correlation coefficients (r^2^) between assay for purple rice extracts.
