# Supplementary material for: Anthocyanin Profile, Antioxidant, Anti-Inflammatory, and Antimicrobial against Foodborne Pathogens Activities of Purple Rice Cultivars in Northern Thailand
Source: Molecules. 2021 Aug 29;26(17):5234. doi: 10.3390/molecules26175234 (PMC8433650; doi:10.3390/molecules26175234)
Supplement: Supplementary file 1 [file molecules-26-05234-s001.zip › molecules-1338100-SI.pdf]

molecules

Supplementary Material

Anthocyanin Profile, Antioxidant, Anti-Inflammatory, and Antimicrobial against Foodborne Pathogens Activities of Purple Rice Cultivars in Northern Thailand

Thanawat Pattananandecha [1,2], Sutasinee Apichai [1,2,3], Sasithorn Sirilun [2], Jakaphun Julsrigival [1,2], Kasirawat Sawangrat [1,2], Fumihiko Ogata [4], Naohito Kawasaki [4,5], Busaban Sirithunyalug [1,2,*] and Chalermpong Saenjum [1,2,3,*]

[1] Cluster of Excellence on Biodiversity Based Economics and Society (B.BES-CMU), Chiang Mai University, Chiang Mai 50200, Thailand; thanawat.pdecha@gmail.com (T.P.); sutasinee.apichai@gmail.com (S.A.); jakaphun@gmail.com (J.J.); kasirawat.s@cmu.ac.th (K.S.)
[2] Department of Pharmaceutical Sciences, Faculty of Pharmacy, Chiang Mai University, Chiang Mai 50200, Thailand; ssirilun@gmail.com
[3] Center of Excellence for Innovation in Analytical Science and Technology (I-ANALY-S-T), Faculty of Science, Chiang Mai University, Chiang Mai 50200, Thailand
[4] Faculty of Pharmacy, Kindai University, 3-4-1 Kowakae, Higashi-Osaka, Osaka 577-8502, Japan; ogata@phar.kindai.ac.jp (F.O.); kawasaki@phar.kindai.ac.jp (N.K.)
[5] Antiaging Center, Kindai University, 3-4-1 Kowakae, Higashi-Osaka, Osaka 577-8502, Japan
* Correspondence: busaban.s@cmu.ac.th (B.S.); chalermpong.s@cmu.ac.th (C.S.); Tel.: +66-81-990-7971 (B.S.); +66-89-950-4227 (C.S.)

Table S1. Pearson correlation coefficients (r_2) between assay for purple rice extracts.

		TPC	TFC	Cyanidin 3-*O*-glucoside	Peonidin 3-*O*-glucoside	Delphinidin 3-*O*-glucoside
Antioxidant activity	ABTS•+	0.960**	0.935**	0.893**	0.826**	0.916**
	Lipid peroxidation	−0.703**	−0.689**	−0.873**	−0.637*	−0.798**
	O2•−	−0.944**	−0.929**	−0.954**	−0.760**	−0.913**
	NO	−0.786**	−0.765**	−0.923**	−0.710**	−0.838**
Pro-inflammatory protein inhibition	NO	−0.962**	0.879**	−0.868**	−0.822**	−0.762**
	iNOS	−0.940**	0.904**	−0.872**	−0.802**	−0.875**

** Correlation is significant at the 0.01 level (2-tailed).

* Correlation is significant at the 0.05 level (2-tailed).
